# Effects of Pesticide Treatments on Nutrient Levels in Worker Honey Bees (*Apis mellifera*)

**DOI:** 10.3390/insects7010008

**Published:** 2016-03-01

**Authors:** Haley K. Feazel-Orr, Katelyn M. Catalfamo, Carlyle C. Brewster, Richard D. Fell, Troy D. Anderson, Brenna E. Traver

**Affiliations:** 1 Engineering Consulting Services Mid-Atlantic, Limited Liability Company (LLC), 14026 Thunderbolt Place, Suite 100, Chantilly, VA 20151, USA; hkfeazel@vt.edu; 2 Department of Entomology, Virginia Tech, 170 Drillfield Drive, Blacksburg, VA 24061, USA; kmcat92@vt.edu (K.M.C.); carlyleb@vt.edu (C.C.B.); rfell@vt.edu (R.D.F.); anderst@vt.edu (T.D.A.); 3 Biology Department, Penn State Schuylkill, 200 University Drive, Schuylkill Haven, PA 17972, USA

**Keywords:** honey bee, *Apis mellifera*, nutrition, pesticide, colony losses

## Abstract

Honey bee colony loss continues to be an issue and no factor has been singled out as to the cause. In this study, we sought to determine whether two beekeeper-applied pesticide products, *tau*-fluvalinate and Fumagilin-B^®^, and one agrochemical, chlorothalonil, impact the nutrient levels in honey bee workers in a natural colony environment. Treatments were performed in-hive and at three different periods (fall, spring, and summer) over the course of one year. Bees were sampled both at pre-treatment and two and four weeks post-treatment, weighed, and their protein and carbohydrate levels were determined using BCA and anthrone based biochemical assays, respectively. We report that, based on the pesticide concentrations tested, no significant negative impact of the pesticide products was observed on wet weight, protein levels, or carbohydrate levels of bees from treated colonies compared with bees from untreated control colonies.

## 1. Introduction

Honey bees play a key role in the pollination of agricultural crops [1,2], and the recent decline of honey bee populations is thought to pose a threat to the availability of certain foods. Within the last decade, beekeepers in both the U.S. and Europe have experienced a significant increase in colony losses [1]. For the last seven years, U.S. winter losses have averaged 30% [2]. The exact cause of the increased losses is unknown but is thought to be due to the combined stress from parasites, pathogens, and pesticides [2,3]. Pesticides have been targeted as a major factor, causing not only direct losses, but also reductions in honey and wax production and pollination benefits to the tune of over $283 million per year [4]. Annual losses from honey bee pollination of crops alone are estimated at around $210 million [4]. Other possible explanations for declining honey bee populations include loss of habitat, poor nutrition from decreased forage availability, and beekeeping management practices that increase colony stress [3].

The increased use of pesticides in agriculture has exposed honey bees to a continual array of chemicals, including miticides, insecticides, fungicides, and herbicides. As a result, residues of many pesticides have been found in beeswax and pollen, as well as adult and pupal bees [5]. A number of these compounds have also been shown to have sub-lethal effects on bees, causing delayed development, shortened adult longevity, and immune system impairment [6,7]. The miticide *tau*-fluvalinate, fungicide chlorothalonil, and antibiotic Fumagilin-B^®^ (Medivet Pharmaceuticals Ltd., High River, AB, Canada) are thought to trigger sub-lethal effects and impact the honey bee immune system [5]. Though the use of a single pesticide has not been singled out as responsible for honey bee losses, the interaction of these pesticides with pathogens and pests, including nosema and varroa mites, could be a factor in colony decline [8].

*tau*-Fluvalinate is a synthetic pyrethroid that has been commonly used by beekeepers for varroa mite control, and residues are often found in hive products long after treatment due to its lipophilic nature [5]. Commonly sold as Apistan^®^ (Wellmark International, Schaumburg, IL, USA), *tau*-fluvalinate is a miticide, which is tolerated by honey bees [9]. The treatment is applied to plastic strips that are inserted between the combs of a hive, and are left for up to six weeks in the fall to provide constant exposure of the miticide to the mite population [9]. Varroa mites decrease the vigor of the hive by repeated feeding on both developing and adult bees and by acting as a vector for viral diseases, including Kashmir bee virus and deformed wing virus [10]. Chlorothalonil is a widely used agricultural fungicide and one of the most common fungicides used by home owners. Since it is often applied during bloom, honey bees can come into contact with chlorothalonil while foraging and has been frequently identified as a residue in pollen, often at high levels [11]. Fumagilin-B^®^, on the other hand, is an antibiotic used by beekeepers for the prevention and treatment of nosema [12]. Bees are exposed to this antibiotic directly as beekeepers feed Fumagilin-B^®^ to colonies in sugar syrup.

Nutrition is critical to the immune functions of organisms as it allows the organism’s natural responses to react towards toxicants and parasites. In *Drosophila melanogaster,* it has been shown that larvae exposed to limited nutritional resources were more likely to die after being exposed to a parasite compared to those with excess nutritional resources [13]. Moret and Schmid-Hempel [14] showed that activation of the bumblebee immune response can decrease a bee's longevity due to the increased use of resources for immune activity that could be otherwise used to maintain survival. In honey bees, nutritional levels have been shown to affect adult longevity, brood-food gland development, and sensitivity to pathogens and pesticides [15,16,17,18]. Diet protein quality, as measured by pollen diversity, has also been shown to enhance both individual and social immunocompetence in honey bees. Proper nutrition allows organisms to have increased tolerance to various stressors by providing adequate resources for coping with these stressors, whereas those organisms without proper nutrition are more likely to succumb to stressors [17,19].

The purpose of this study was to determine whether commonly encountered agrochemicals, such as chlorothalonil, and the pesticide products Fumagilin-B^®^ and Apistan^®^ (*tau*-fluvalinate) affect nutrient levels in honey bees. This study was part of a larger project to determine whether the pesticides listed above impact other factors such as honey bee pathogen loads and immunity. Our study was performed in-hive instead of in cages to examine the effect that these materials have on bees in a more natural colony environment. Although the use of in-hive testing limits our ability to control extraneous factors, such as age, individual pesticide consumption, the bee’s initial nutritional status, this approach provides a better understanding of a colony level response and how the materials might affect overall hive health as analyzed by bee nutrient status. By analyzing the effect of pesticide exposure on bee weight, and protein and carbohydrate levels in adult workers, we hoped to determine whether chlorothalonil, Fumagilin-B^®^, or *tau-*fluvalinate might impact bee health through changes in overall nutrient composition.

## 2. Experimental Section

### 2.1. Colony Establishment

Twenty one-story colonies were established utilizing splits with young queens, in the summer of 2012 at two apiaries near Blacksburg, VA. The apiaries were located ~3.2 km apart at locations with similar weather patterns and bee foraging resources. Fifteen of the 20 experimental colonies randomly received one of three chemical treatments, Fumagilin-B^®^, *tau*-fluvalinate (Apistan^®^ strips), or chlorothalonil, with five colonies treated with each pesticide product; the remaining five colonies served as untreated controls and did not receive any pesticide treatment. The treatment hives were divided equally between the two apiaries. Treatments were applied once in each of three seasons during the study—fall (October 2012), spring (April 2013), and summer (July 2013). Each of the experimental colonies received the same treatment throughout the study, and any colony that was lost was not replaced. For example, all colonies that received a Fumagilin-B^®^ treatment in the fall received a Fumagilin-B^®^ treatment during subsequent periods (*i.e.*, spring and summer).

All pesticide treatments, with the exception of chlorothalonil, were administered as beekeepers would apply them and at the recommended dosage. Colonies treated with *tau-*fluvalinate received 2 Apistan^®^ strips, as per manufacturer’s instructions, and 3.8 L of 50% sugar syrup. Fumagilin-B^®^-treated colonies received 5 grams of Fumagilin-B^®^ dissolved in 3.8 L 50% sugar syrup as per manufacturer’s instructions. Chlorothalonil-treated colonies received 3.8 L of 50% sugar syrup that had a final concentration of 10 parts per billion (ppb, which is 10 μg/L) of chlorothalonil. The chlorothalonil concentration was determined using the median level of chlorothalonil residues found in bees reported by Mullin *et al.* [5] and the mean levels of chlorothalonil residues in stored pollen samples reported by Bernal *et al.* [20]. The five untreated control colonies received 3.8 L of 50% sugar syrup to minimize differences due to feeding.

From each colony, a random sample of 30–40 worker bees was collected from an area near the brood nest for each of the pesticide-treated and control colonies prior to treatment (pre-treatment), and at 2 and 4 weeks post-treatment during each of the three seasons. The samples were stored on ice in the field and then transferred to a freezer at −80 °C. Fifteen individual bees were then selected from each sample for examination of total wet weight and nutrient status, as measured by total protein and carbohydrate levels. Sampling was conducted at 2 and 4 weeks post-treatment because the study was part of a larger project designed to examine pesticide effects on pathogen (virus and *Nosema* spp.) levels in which bees were collected at these times based on the infection cycle of *Nosema* spp.

### 2.2. Biochemical Assays

Biochemical assays were performed utilizing methods adapted from Van Handel’s nutritional studies in mosquitoes [21,22] and from a study with honey bees [23]. For protein and carbohydrate extraction, individual honey bees were placed in 1.5 mL microfuge tubes and manually crushed using a plastic pestle in 300 μL of 100 mM sodium phosphate buffer (pH 7.8). Samples were centrifuged at 16,000 rpm for 15 min at 4 °C. The supernatant was removed and used for analysis. Protein samples were diluted 1:50 and carbohydrate samples were diluted 1:25 in the sodium phosphate buffer before analysis. All assays were performed on a Molecular Devices Microplate Reader (Sunnyvale, CA, USA). Protein levels were determined using a BCA assay following the manufacturer’s directions (Sigma Aldrich, St. Louis, MO, USA) with absorbance readings at 595 nm and quantified by comparison to BSA standards. Anthrone-based assays from Van Handel [21,22] were used to measure carbohydrate levels, and samples were read at 620 nm. Total carbohydrate levels were determined from glucose standards. All samples were read in triplicate and normalized to the wet weight of the honey bee.

### 2.3. Statistical Analysis

Individual analyses were conducted on the data collected during each season (fall, spring, and summer) to determine the effects of pesticide treatment and sampling time (0, 2, and 4 weeks) on each of the three response variables (bee wet weight, protein level, and carbohydrate level). Because the experimental design for the study was hierarchical, *i.e.*, bees sampled from hives nested within treatments [24], and data collection involved repeated measurements of the response variables over time, the data were analyzed using a linear mixed model for repeated measures (LMMRM) analysis [25,26,27,28] rather than by repeated measures ANOVA or MANOVA [26]. The LMMRM approach has advantages over repeated measures ANOVA and MANOVA in that it can accommodate unbalanced data (*i.e.*, experimental units with missing data) and does not require that the data have a covariance structure that satisfies the sphericity assumption (ANOVA) or is unstructured (MANOVA), but it provides the flexibility to define a covariance structure appropriate for the data [25,26]. As recommended by Littell *et al.* [25], we used a first-order autoregressive with random effect covariance structure for the LMMRM analyses with treatment, sampling time, and their interaction as fixed effects factors, hive nested within treatment as the random effects factor, and sampling time and hive as the repeated measures parameter and subject, respectively. Preliminary analysis showed that the apiary did not have a significant effect on the results, so the data from the two apiaries were combined. Prior to each analysis, the response variable was tested for normality and, where necessary, was transformed using a Box-Cox transformation [29]. All statistical analyses were carried out using JMP Pro 11.0.0 (SAS Institute, Cary, NC, USA, 2013) at a significance level of α = 0.05.

## 3. Results

Four of the 20 experimental colonies died during the study. Three of the four colonies died during the winter and spring of 2013. The cause of colony mortality was diagnosed as starvation due to the presence of dead bees in a well-formed cluster and no honey in the vicinity. Each colony had several frames of brood at the center of the cluster and frames of honey in the hive, but a period of cold temperatures prevented access to the honey stores. Two of these colonies had been treated with chlorothalonil in the fall 2012 while the other had been treated with *tau*-fluvalinate. The fourth colony, treated with chlorothalonil, was observed to be dwindling prior to the summer treatment in July 2013 and died shortly thereafter.

**Figure 1:**
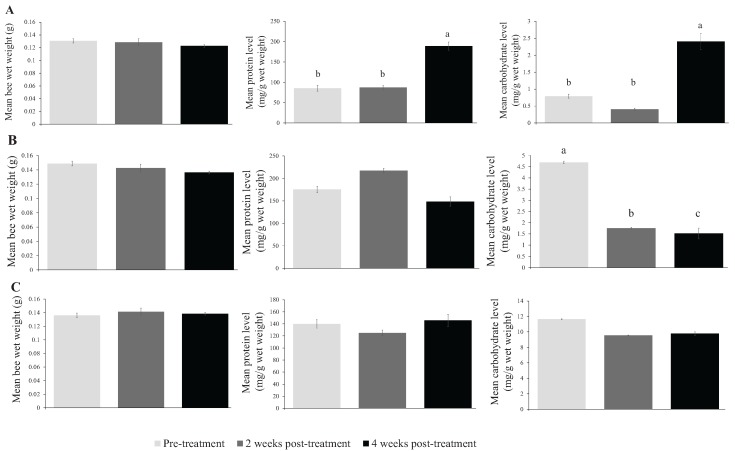
Mean bee wet weight, protein levels, and carbohydrate levels of bees samples from all colonies, regardless of treatment, at three sampling time points during three seasons (fall, spring, and summer). Pesticide treatment had no significant effect on the variables examined, so the data were pooled across the different treatments (three pesticides and control, untreated colonies) for each sampling period within each season (**A**) fall 2012 (**B**) spring 2013 (**C**) summer 2013. Significant differences are indicated by different lowercase letters (α = 0.05).

Overall, the results show that, compared with the control untreated colonies, the pesticide treatments, *tau*-fluvalinate, chlorothalonil, and Fumagilin-B^®^ did not have a significant effect on the wet weight, protein, or carbohydrate levels of honey bees, at the concentrations tested, when examined by season (Table 1). In the fall, there were no significant differences in bee wet weights with regard to treatment (*F*_3, 9.9_ = 2.123, *p* > 0.05), sampling time (*F*_2, 18.0_ = 0.724, *p* > 0.05) (Table 2; Figure 1A), or the interaction of treatment and sampling time (*F*_6, 17.8_ = 1.023, *p* > 0.05). Although there were no significant treatment (Table 1) or interaction effects on protein (treatment: *F*_3, 10.2_ = 0.182, *p* > 0.05; interaction: *F*_6, 17.1_ = 0.237, *p* > 0.05) and carbohydrate (treatment: *F*_3, 7.8_ = 0.317, *p* > 0.05; interaction: *F*_6, 20.0_ = 0.369, *p* > 0.05), the levels of these macromolecules differed significantly among the three sampling times (protein: *F*_2, 17.2_ = 6.55, *p* < 0.01; carbohydrate: *F*_2, 20.1_ = 9.274, *p* < 0.01) with the mean level at 4-weeks post-treatment significantly higher compared with the levels at pre-treatment and 2 weeks post-treatment (Table 2; Figure 1A).

In the spring, there was no significant effect of treatment (*F*_3, 11.1_ = 0.654, *p* > 0.05), sampling time (*F*_2, 22.0_ = 1.788, *p* > 0.05), or their interaction (*F*_6, 22.3_ = 0.942, *p* > 0.05) on bee wet weights (Table 1; Figure 1B). Similar results were obtained for total protein levels in bees (treatment: *F*_3, 14.6_ = 1.043, *p* > 0.05; sampling time: *F*_2, 27.5_ = 1.931, *p* > 0.05; interaction: *F*_6, 26.9_ = 0.796, *p* > 0.05) (Table 1 and Table 2; Figure 1B). There were also no significant treatment (*F*_3, 11.6_ = 0.512, *p* > 0.05) (Table 1) or interaction (*F*_6, 18.8_ = 2.281, *p* > 0.05) effects on total carbohydrate levels during the spring; however, carbohydrate levels differed significantly among the three sampling times (*F*_2, 18.6_ = 24.247, *p* > 0.05). Mean carbohydrate levels were significantly higher at pre-treatment sampling, but decreased significantly at the 2-week and 4-week post-treatment samplings (Table 2; Figure 1B).

Finally, during the summer, no significant differences in bee wet weight were observed with respect to treatment (*F*_3, 12.8_ = 0.404, *p* > 0.05), sampling time (*F*_2, 22.8_ = 0.446, *p* > 0.05), and their interaction (*F*_6, 23.2_ = 0.704, *p* > 0.05) (Table 1; Figure 1C). Similar results were observed for the levels of two macromolecules, protein (treatment: *F*_3, 12_ = 0.632, *p* > 0.05; sampling period: *F*_2, 24_ = 0.265, *p* > 0.05; interaction: *F*_6, 24_ = 0.993, *p* > 0.05) and carbohydrate (treatment: *F*_3, 7.4_ = 0.645, *p* > 0.05; sampling period: *F*_2, 15_ = 0.933, *p* > 0.05; interaction: *F*_6, 15.6_ = 2.750, *p* > 0.05) (Table 1 and Table 2; Figure 1C).

## 4. Discussion

Honey bee physiology varies at different times of the year and leads to seasonal differences in the carbohydrate, lipid, and protein content of workers [30,31,32,33]. These differences could affect how workers respond to low levels of pesticide exposure, particularly materials used by beekeepers to control hive parasites such as varroa mites or *Nosema ceranae*. In this study, however, the treatment of colonies with *tau*-fluvalinate, Fumagilin-B^®^ or chlorothalonil at three different times during the year did not affect the worker conditions examined. Bee wet weight and total protein and total carbohydrate levels in treated and control honey bees did not differ significantly between treated and untreated bees, suggesting that the pesticide treatments did not affect honey bee nutrient levels at the concentrations tested. The variance observed in the data related mainly to sampling time and not to the effects of individual treatments.

All statistical comparisons were made within seasons and not between seasons because of changes in hive number and identity caused by colony losses. No differences were found among bee wet weights with respect to any of the sampling times. The mean weight of sampled workers varied from a low of 123 mg in fall-collected bees to a high of 149 mg in spring-collected bees. This finding is not surprising since bee weights do not generally change much throughout the year, regardless of physiological changes that take place during different seasons [30].

During fall 2012, significant differences were observed in protein levels between bees collected during the pre-treatment and two weeks post-treatment periods and those collected during the four week post-treatment sampling period. These differences can most likely be attributed to physiological changes associated with the development of winter bees. Worker bees reared in the fall must carry the colony through the winter and are characterized by increased longevity with life spans of three to eight months. Winter bees remain physiologically young and are similar to nurse bees in summer, having well developed hypopharyngeal glands and higher protein levels in the hemolymph and fat bodies [34]. In Virginia, brood rearing typically continues into early November, such that during the fall, the proportion of summer bees declines, as the population of winter bees increases. By mid-November, when the last group of samples was collected, hive populations consisted primarily of long-lived winter bees. The mean protein level for the bees collected in mid-November was 23.2 mg/bee (18.9% of fresh body weight), which was similar to the protein levels in winter bees collected the first week of January from the same hives (21.9 mg/bee; 16.9% of fresh weight) [35]. Since brood rearing in southwest Virginia typically does not start until mid to late January, declines in worker protein levels would not be expected until the resumption of brood rearing [30,33].

The protein levels of worker bees in the spring are consistent with higher nurse bee populations, resulting from high levels of brood rearing and high colony growth rates. However, no differences were found among protein levels for any of the pre-treatment or post-treatment groups. Lower protein levels are generally expected for workers collected in mid-summer [30,31], but again, no evidence of treatment effects was observed among pre- and post-treatment samples.

Although carbohydrate levels were generally low during the fall, there was a small increase in these levels in the four-week post-treatment bees when compared to both the pre-treatment and two-week post-treatment bees. This difference was not due to treatment effects and might be explained by the hive transition from summer to winter bees and the initiation of clustering behavior as outside temperatures declined. Shehata *et al.* [30], for example, found that worker hemolymph sugar levels declined from October to November and then increased during the following months. However, carbohydrate levels in the fat body of workers decreased during the winter and increased during spring and summer. Total carbohydrate levels in this study were lowest in fall and winter collected bees [35], increasing to the highest levels in bees sampled when colonies were actively foraging during the spring and summer. Low carbohydrate levels in late fall and winter are not unexpected due to the need for endothermic heat production by shivering [36]. Significant differences were noted in the spring carbohydrate levels of pre-treatment bees compared to the post-treatment groups, but the differences were not due to treatment effects since similar responses were noted in bees from the control hives. No differences were observed between pretreatment and post-treatment groups during any of the seasonal periods that could be associated with pesticide treatment. These findings suggest that none of the pesticidescause significant impacts at the levels tested with respect to honey bee weight or nutrient levels, even when exposed over an extended period.

While there has been discussion about how pesticides impact the health of honey bee colonies, our study suggests that two beekeeper-applied pesticides, *tau*-fluvalinate and Fumigilin-B^®^, and the commonly encountered fungicide chlorothalonil, at recommended or naturally occurring concentrations, do not negatively impact the health of honey bees, as measured by protein and carbohydrate nutrient levels.

## Figures and Tables

**Table 1:** Mean (±standard error) wet weight and macromolecule levels for worker honey bees treated with the one of four treatments (three pesticides and a control) in the fall, spring, and summer.

Season (Year)	Treatment	Wet Weight (g)	Protein Level (mg/g)	Carbohydrate Level (mg/g)	N ^1^
Fall 2012	Control	0.118 ± 0.002	92.0 ± 5.54	1.306 ± 0.214	121
	Chlorothalonil	0.137 ± 0.005	118.7 ± 10.7	0.956 ± 0.131	158
	Fumagilin	0.119 ± 0.002	115.27 ± 8.0	1.130 ± 0.157	155
	*tau*-Fluvalinate	0.136 ± 0.007	122.3 ± 10.4	0.963 ± 0.01	141
Spring 2013	Control	0.146 ± 0.004	177.2 ± 8.7	2.117 ± 0.184	213
	Chlorothalonil	0.138 ± 0.003	181.4 ± 11.2	1.625 ± 0.245	92
	Fumagilin	0.141 ± 0.002	168.0 ± 9.1	3.012 ± 0.326	173
	*tau*-Fluvalinate	0.141 ± 0.004	189.9 ± 10.8	3.318 ± 0.307	169
Summer 2013	Control	0.135 ± 0.002	116.8 ± 6.7	7.49 ± 0.476	209
	Chlorothalonil	0.142 ± 0.002	142.2 ± 8.9	7.5 ± 0.557	75
	Fumagilin	0.139 ± 0.002	159.8 ± 8.1	13.94 ± 0.973	222
	*tau*-Fluvalinate	0.139 ± 0.002	129.4 ± 7.3	8.204 ± 1.563	179

^1^ Number of individual bees examined at each sampling time.

**Table 2:** Mean (±standard error) wet weight and macromolecule levels combined across four treatments (three pesticides and a control) for worker honey bees tested in fall, spring, and summer.

Season (Year)	Sampling Time	Wet Weight (g)	Protein Level (mg/g)	Carbohydrate Level (mg/g)	N ^1^
Fall 2012	Pre-treatment	0.131 ± 0.003	283.9 ± 23.8 ^b^	2.635 ± 0.193 ^b^	228
	2 weeks post-treatment	0.128 ± 0.005	291.6 ± 15.9 ^b^	1.343 ± 0.124 ^b^	197
	4 weeks post-treatment	0.123 ± 0.002	629.6 ± 34.0 ^a^	8.009 ± 0.799 ^a^	150
Spring 2013	Pre-treatment	0.149 ± 0.003	585.0 ± 22.0	15.633 ± 0.768 ^a^	207
	2 weeks post-treatment	0.143 ± 0.003	725.3 ± 36.1	5.859 ± 0.505 ^b^	201
	4 weeks post-treatment	0.136 ± 0.003	495.2 ± 24.9	5.067 ± 0.839 ^c^	239
Summer 2013	Pre-treatment	0.136 ± 0.002	386.2 ± 21.2	38.933 ± 3.689	207
	2 weeks post-treatment	0.141 ± 0.002	345.4 ± 18.8	31.824 ± 1.133	238
	4 weeks post-treatment	0.138 ± 0.001	446.4 ± 25.0	32.594 ± 1.744	240

^1^ Number of individual bees examined at each sampling time. Different lowercase letters within each season indicate significant differences (*p* < 0.05).
